# Persistence of sugars used for intestinal permeability measures in an in vitro rumen environment

**DOI:** 10.3168/jdsc.2021-0202

**Published:** 2022-04-26

**Authors:** M.D. Ellett, C.L.M. Parsons, J.M. Hay, K.M. Daniels

**Affiliations:** 1 Dairy Science Department, Virginia Polytechnic Institute and State University, Blacksburg 24061; 2 ECC Test Lab, 10329 Stony Run Ln., Ashland, VA 23005

## Abstract

Orally administered synthetic sugars are routinely used as markers of intestinal permeability in nonruminants and young calves, but not adult ruminants, likely because of uncertainty surrounding degradation of such sugar markers (e.g., d-mannitol, sucralose, lactulose) in the rumen. The objective was to evaluate persistence of d-mannitol, sucralose, and lactulose in a closed in vitro rumen fermentation system over 48 h. The null hypothesis was that sugar concentration would not be affected by time. Rumen contents were collected and processed under anerobic conditions a total of 12 times from a ruminally cannulated lactating Holstein cow. These 12 rumen samplings reflect 4 in vitro experiments (d-mannitol, sucralose, lactulose, and d-glucose as a positive methodological control), each replicated 3 times. For each replication, filtered rumen contents and rumen buffer (1:3; vol/vol) were added to a series of six 500-mL flasks, each containing 3 filter bags. Each filter bag contained 500 mg of ground total mixed ration (94.2% dry matter; 15.2% crude protein, 40.9% neutral detergent fiber, 3.9% fat, and 6.2% ash, dry matter basis) and three 5-mm glass beads. The 6 flasks represented 0, 6, 12, 24, and 48 h time points, and a 48-h negative control flask. A single sugar was tested during each experimental replicate. Final flask concentrations of each sugar were 4.07 mg/mL d-glucose, 1.99 mg/mL d-mannitol, 2.17 mg/mL sucralose, or 3.10 mg/mL lactulose. Flasks were incubated under anerobic conditions at 39°C where they remained undisturbed until the designated time of removal (0, 6, 12, 24, or 48 h). At removal, an aliquot of each flask was removed and sugar concentration was quantified by HPLC-mass spectrometry. Data for each experiment were analyzed using an ANOVA model that included the single fixed effect of time (0, 6, 12, 24, or 48 h); flask within replicate was the random term. Lactulose was not resolved in any samples due to interfering components within the sample matrix; no lactulose data are presented. As expected, positive methodological control of glucose decreased to negligible concentrations by 6 h of in vitro incubation. d-Mannitol followed the same pattern as glucose, which was different from our hypothesis. The interpretation is that d-mannitol is degraded in the in vitro rumen culture system and, by extension, is therefore not a viable choice to use in in vivo intestinal permeability tests in adult ruminants when dosed orally. As hypothesized, sucralose concentration did not change over 48 h of incubation in a closed in vitro rumen fermentation system. This suggests feasibility of orally dosed sucralose in adult ruminants as a rumen-inert marker of intestinal permeability with subsequent analysis of biological samples (e.g., urine, blood) by HPLC-mass spectrometry.

## Highlights

• Sucralose persisted in an in vitro rumen environment for 48 h.
• d-Glucose and d-mannitol were nondetectable at 6 h in an in vitro rumen environment.
• Lactulose could not be quantified in the rumen content and medium matrix via high-performance liquid chromatography-mass spectrometry.
• Neutral detergent fiber loss and the medium pH were unaffected by treatment.

## Graphical Abstract

**Figure:**
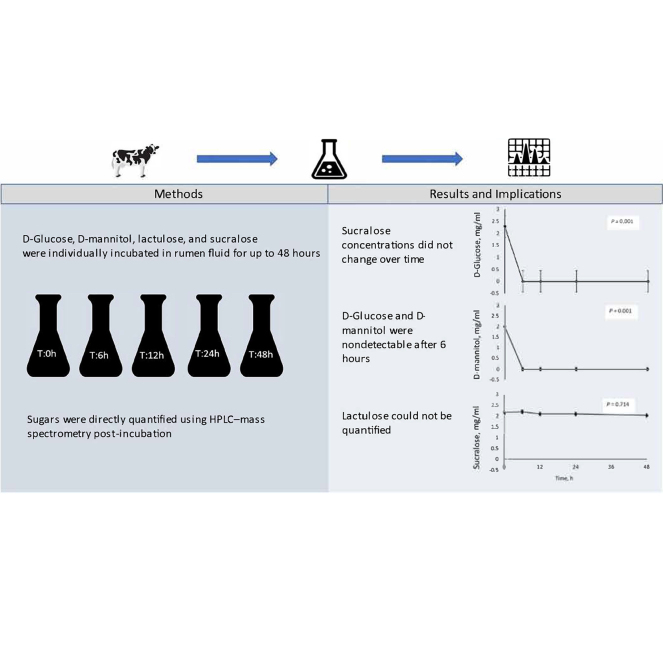
**Summary:** Sucralose, d-mannitol, and lactulose are synthetic sugars commonly used for intestinal permeability measures in monogastric and preruminant animals. These synthetic sugars are not used in adult ruminants due to uncertainty pertaining to their stability within the rumen. When exposed to an in vitro rumen environment, d-mannitol was depleted at a rate comparable to that of the methodological control, d-glucose. Sucralose concentrations did not change after 48 h of exposure. Lactulose could not be quantified within the rumen content and media mixture via high-performance liquid chromatography-mass spectrometry.

Synthetic sugars have been used by scientists and physicians for many years as markers of intestinal permeability in nonruminants (Rao et al., 2011; Wijtten et al., 2011), newborn calves (Klein et al., 2007; Araujo et al., 2015), and peri-ruminant calves (Amado et al., 2019). Use in adult ruminants is less documented, likely because of uncertainty surrounding “inertness” of such sugar markers (e.g., lactulose, mannitol, sucralose) in the rumen.

In the only known study of its kind, Ahmed et al. (2013) systematically added naturally occurring (e.g., glucose, fructose, mannitol) and synthetic (e.g., lactulose, sucralose) sugars to an in vitro rumen fermentation system. Rumen inoculum was obtained from rumen-cannulated heifers fed an 85% forage, 15% concentrate ration (DM basis; Ahmed et al., 2013). Gas pressure measurements and VFA production were assessed over a period of 24 h (Ahmed et al., 2013). The authors found that lactulose and d-mannitol were fermented to a lesser extent than sugars commonly found in feedstuffs, such as fructose; sucralose was not fermented (Ahmed et al., 2013). Direct measurement of sugar molecules in rumen contents by either liquid or gas chromatography was not included in previous work and, to our knowledge, is not yet reported in the literature. Direct quantification of sugar molecules after exposure to the rumen environment will elucidate the persistence of the marker. The in vivo use of sugar markers for intestinal permeability studies in adult ruminants may be ill advised until it is shown that the markers will not be degraded within the rumen.

The aim of the present work was to use HPLC-MS to directly quantify the amount of select sugars present in a closed in vitro rumen fermentation system at various time points over 48 h of anerobic incubation. The expected novel outcome was that any sugars remaining in the system after 48 h of incubation, could be candidate rumen-inert markers, likely suitable for in vivo oral dosing and use as markers of leaky gut in mature ruminants. Sugars were d-mannitol, lactulose, sucralose, and d-glucose (positive methodological control). We hypothesized that quantities of sucralose and lactulose (both synthetic sugars), and the sugar-alcohol d-mannitol do not change over time, whereas we expected quantity of d-glucose to decrease to nondetectable levels within 48 h of in vitro incubation with rumen contents.

The Virginia Tech Institutional Animal Care and Use Committee approved the in vivo procedures related to rumen content removal from a single lactating and ruminally cannulated Holstein cow (protocol #19–061). The cow was fed a corn-silage based TMR balanced for stage of lactation. Rumen content collection and processing occurred a total of 12 times between October and December of 2020 with at least 48 h of rest between samplings. These 12 rumen samplings reflect 4 in vitro experiments, each replicated 3 times.

At each rumen sampling and approximately 3 h before scheduled animal feeding, approximately 2 L of rumen contents was collected through the cannula and processed as follows. First, 500 mL of rumen contents, consisting of a heterogeneous mixture of rumen fluid and solids, was aliquoted and stored in a prewarmed (39°C), airtight plastic bag and placed in an insulated cooler filled partially with 39°C water. Second, approximately 1.5 L of rumen contents was filtered (hand-squeezed) through 4 layers of cheesecloth until 1.0 L of filtrate was obtained. Filtrate was collected into a prewarmed (39°C) plastic bottle. The bottle was then purged of oxygen by squeezing until contents overflowed. As soon as the contents overflowed, the lid was placed back onto the plastic bottle and tightened. The bottle was then placed in the insulated cooler for transit. Previous steps were performed within 3 min of removal from the rumen. Samples were then transported to a research laboratory at Virginia Tech (~30 min for transit).

Immediately upon arrival to the laboratory, the 500 mL of rumen contents was recombined with 1.0 L of rumen filtrate and blended for 30 s in anaerobic conditions (95% CO_2_, 5% O_2_) at 39°C in a modified commercial blender (Waring). After blending, the total contents of the blender were filtered through 4 layers of cheese cloth. Next, 500 mL of the resulting filtrate was added to 1,500 mL of preheated rumen buffer medium (vol/vol) to achieve a final volume of 2 L. The rumen buffer medium was prepared as described by Ankom Procedures (2020) and mainly contained 17.5% sodium carbonate, 2.5% potassium phosphate, and 1.25% sodium sulfide hydrate in water.

From this 2-L stock of rumen inoculum, one 250-mL aliquot was removed and added to one 500-mL glass Erlenmeyer flask; this flask contained no added sugars and was designated the negative control. All Erlenmeyer flasks were prefilled with 3 heat-sealed F57 filter bags (Ankom Technology); each filter bag contained 500 mg of dried and ground TMR from a single stock (94.2% DM; 15.2% CP, 40.9% NDF, 3.9% fat, 6.2% ash, 33.8% NFC, DM basis) and three 5-mm glass beads (Thermo Fisher Scientific). The TMR provided nutrients for the microbes and the glass beads submerged the filter bags in the rumen inoculum, thereby preventing surface floating.

A single sugar solution, containing either glucose (experiment 1), mannitol (experiment 2), sucralose (experiment 3), or lactulose (experiment 4) was added to the remaining 1,750 mL of rumen inoculum stock, as detailed next. The sugar to be tested within replicate was drawn at random from a set of 4 labeled tubes; after removal, the labeled tube was not replaced. This randomization process was repeated a total of 3 times by author MDE. For each glucose experiment replicate, a total of 4.07 g of glucose (d-glucose; Sigma-Aldrich) was dissolved into distilled water and brought to a final volume of 35 mL. This was added to the 1,750 mL of rumen inoculum. Thus, the final concentration of glucose used in experiments was 2.28 mg of d-glucose/mL (4,070 mg of glucose/1,785 mL; wt/vol). The same general procedure was used for the other sugars to yield final concentrations of 1.99 mg of d-mannitol/mL (d-mannitol; Acros Organics), 2.17 mg of sucralose/mL (sucralose; Spectrum Chemical MFG Corp.), and 3.1 mg of lactulose/mL (lactulose; Spectrum Chemical MFG Corp.). The concentrations we used were selected to match potential in vivo dosages, where documented. For instance, when orally dosed for intestinal permeability research, d-mannitol has been used at 90 to 120 mg/kg BW (preruminant calves; Klein et al., 2007; Araujo et al., 2015; and Amado et al., 2019) and lactulose at 235 to 450 mg/kg BW (preruminant calves; Klein et al., 2007; Araujo et al., 2015; and Amado et al., 2019); however, these doses are not rigid. In the case of sucralose, dose was determined based on its molecular weight similarity to lactulose (397.6 and 342.1 g/mol, respectively) to arrive at an approximate in vivo dose of 400 mg/kg BW. If orally dosed to adult ruminants, sugars would first be deposited in the reticulo-rumen, where they may or may not undergo microbial degradation before passing to the small intestine. We assumed a 595-kg dairy cow to have approximately 95 L of reticulo-rumen contents (Oba and Allen, 2000; Voelker and Allen, 2003). So, in a theoretical in vivo intestinal permeability study with an intended dose of 318 mg of d-mannitol/kg of BW, a 595-kg cow would be dosed with 189 g of d-mannitol. The 189 g of d-mannitol would enter into approximately 95 L of reticulo-rumen contents, yielding 2.00 mg of d-mannitol/mL of reticulo-rumen contents, which is approximately the concentration we used in our in vitro rumen culture system. Similar math was used to arrive at in vitro concentrations of sucralose, lactulose, and glucose.

Within each replicate, after the designated sugar solution was added to the rumen inoculum, five 250-mL aliquots were removed and added to five 500-mL Erlenmeyer flasks, each containing 3 heat-sealed F57 filter bags, as detailed above. Flasks were purged with 95% CO_2_ and 5% O_2_, fitted with a fermentation lid, and placed in a 39°C orbital shaking water bath set to 125 oscillations/min where they remained undisturbed until the designated time of removal (0, 6, 12, 24, or 48 h). At the designated time of removal, 1 of these 5 flasks was removed at random and labeled appropriately.

The endpoint of 48 h was selected due to its common use in in vitro rumen fermentation procedures. At flask removal, the pH of the contents was recorded using a digital pH meter, and a 25-mL aliquot of the inoculum was removed and placed into a 50-mL conical vial and immediately frozen and stored at −20°C for later analysis of sugar content by HPLC-MS. At a later date, the 25-mL aliquots were shipped frozen to ECC Test Lab (Ashland, VA). To prepare for HPLC-MS, samples were allowed to thaw at room temperature, were vortexed for 30 s, and then centrifuged for 5 min at 3,645 × *g* at 21°C. An aliquot from each sample was removed and diluted to 300 to 400 ppb of the reported starting concentration of each sugar to be assayed (i.e., glucose, mannitol, sucralose, lactulose); the diluent for this was water in 50:50 mobile phase. Samples were run on a triple quadropole for liquid chromatography machine [Agilent Triple Quad MS (LC/TQ); Agilent] using a hydrophilic interaction liquid chromatography column (Agilent Infinity Lab Poroshell 120 HILIC; Agilent). In all, 15 μL of sample was injected onto the Hilic column and eluted with an isocratic mixture of 90% acetonitrile (mobile phase A) and 10% aqueous ammonium hydroxide over the course of a 5-min run. The commercial laboratory used a prepared series of standards for each sugar using analytical grade reagents. d-Glucose, d-mannitol, and sucralose were detected and identified by their retention times and mass spectral analysis. Resultant data from each flask were back-transformed to reflect the concentration of sugar present in each culture flask (milligrams of sugar per milliliter of rumen inoculum). Negative control flask sugar concentrations all registered 0 mg/mL for each experimental replicate (data not shown). The limit of detection for d-glucose, sucralose, and d-mannitol was 100 ppb. The limit of quantification for d-glucose, sucralose, and d-mannitol was 25 ppb.

As a means to assess in vitro rumen culture viability over time, the 3 F57 filter bags contained in the 0, 24, and 48 h flasks for each replicate were retrieved at the designated time, rinsed with cold tap water, and stored at −20°C. Later, bags were thawed at room temperature and NDF quantity per bag was determined using a fiber analyzer (Ankom220, Ankom Technology Corp.) as described by Ferreira and Mertens (2007). Data in Figure 2 are presented as residual NDF percentage. To calculate these values we first determined the amount of NDF in each bag at 0 h, using the weight of TMR added to each bag (DM basis) multiplied by the NDF percentage of 40.9% (DM basis); we called this “beginning NDF.” Then we divided the residual NDF amount, determined using the fiber analyzer, by the beginning NDF for coinciding time points and multiplied by 100, as shown in the equation below.

$$\begin{array}{l} ResidualNDF,\%=\\ \left(\frac{residualNDFfor0,24,or48hflasks}{beginningNDFfor0,24,or48hflasks}\right)\times 100. \end{array}$$

A total of 4 experiments were conducted (glucose, mannitol, lactulose, or sucralose experiment), each repeated 3 times. Each experimental replicate contained 6 flasks. These represented 0, 6, 12, 24, or 48 h time point flasks for the individual sugar being tested and a single negative control flask to which no sugar was added (removed at 48 h time point), respectively (Figure 1). We considered flask within replicate as the experimental unit to which treatment was applied. The MIXED procedure in SAS was used (released 9.4, SAS Institute Inc.) for statistical analysis. Data for each experiment were analyzed separately using an ANOVA model that included the single fixed effect of time (0, 6, 12, 24, or 48 h); flask within replicate was the random term used to test time (10 denominator degrees of freedom; ddfm). Each experiment contained 15 unique observations. Depending on the dependent variable being tested (sugar concentration, pH, or residual NDF), the null hypothesis was that sugar concentration, or pH of the flask, or residual NDF remained the same over time. The alternative hypothesis was that sugar concentration, or pH of the flask, or residual NDF decreased over time. Data are reported as least squares means and standard errors. Significance was declared when *P* < 0.05. We intended to use orthogonal polynomial contrasts to test for linear, quadratic, and cubic effects over time if the main effect of time was significant. However, due to a lack of degrees of freedom, these means separation tests could not be conducted and we limited our interpretation of data and resultant discussion to the main effect of time.

**Figure 1:**
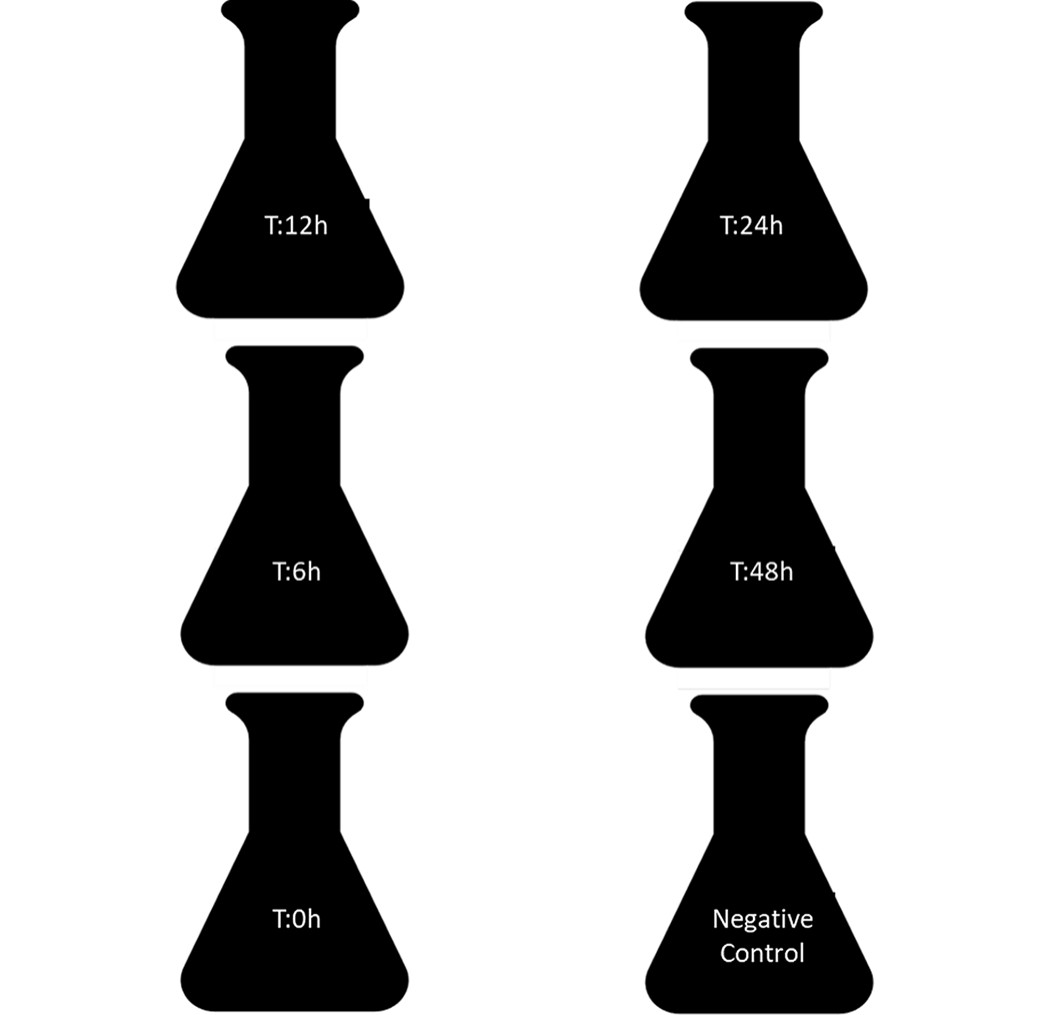
Representation of the flask setup used for each repetition of the experiment. This setup was repeated 3 times for each sugar. Flasks were removed from the water bath at the indicated time (T; 0, 6, 12, 24, or 48 h) and not returned. The negative control flask was removed at 48 h. Upon removal from the water bath, an aliquot was collected from each flask and stored at −20°C for later sugar quantification via HPLC-MS.

Our overall goal was to use a closed in vitro rumen culture system to assess the disappearance of added sugars over the course of 48 h, with the hopes of identifying at least one degradation-resistant sugar. Overall, the in vitro culture system seemed to maintain viability to 48 h, as indicated by the pH and NDF disappearance data in Figure 2. As expected, residual NDF was affected by time (*P* = 0.001).

**Figure 2:**
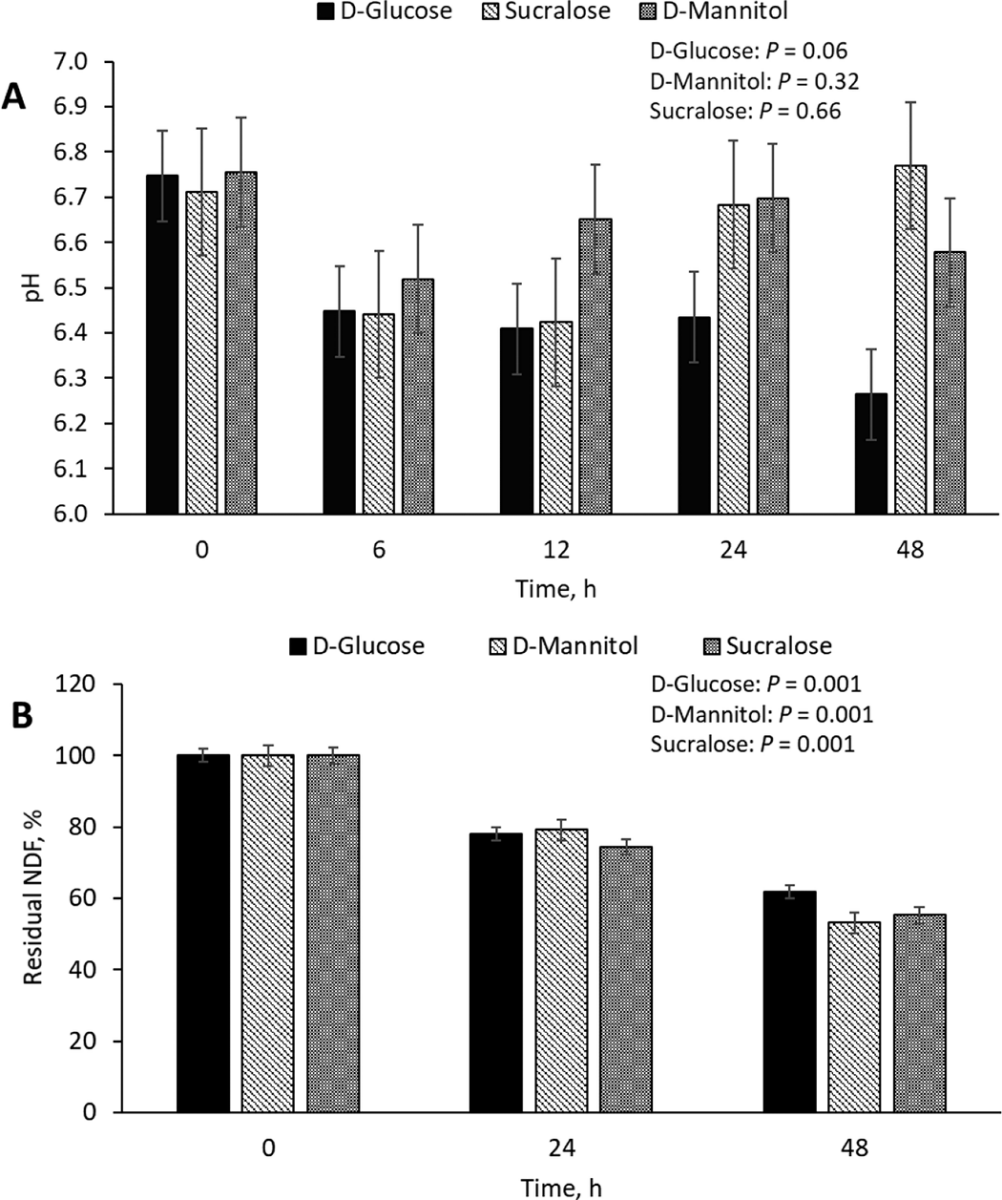
(A) pH in culture flasks over time (0, 6, 12, 24, and 48 h) in a closed in vitro rumen culture system by sugar experiment. Each bar represents the mean of 3 experimental replicates ± SEM. (B) Residual NDF percentage over time (0, 24, 48 h). The NDF content of filter bag residue (n = 3 filter bags per replicate; 3 replicates per experiment) determined by NDF assay. Residual NDF calculated as residual NDF at 0, 24, or 48 h divided by beginning NDF at 0, 24, or 48 h, multiplied by 100.

Figure 3 shows d-glucose, d-mannitol, and sucralose concentrations over time. Lactulose was not resolved in any of the samples by liquid chromatography due to interfering components within the sample matrix; no lactulose data are presented and we suggest caution in using lactulose as a gut permeability marker in adult ruminants for this reason. One major conclusion of our work is that we show that when used at biologically appropriate doses, sucralose and d-mannitol, but not lactulose concentrations, can be directly quantified by liquid chromatography in the complex matrix of rumen inoculum. When used as intestinal permeability markers, concentrations of these sugars are either measured in urine or in blood serum or plasma; methods used here could easily be adapted for measurement of these sugars in such alternate sample matrices.

**Figure 3:**
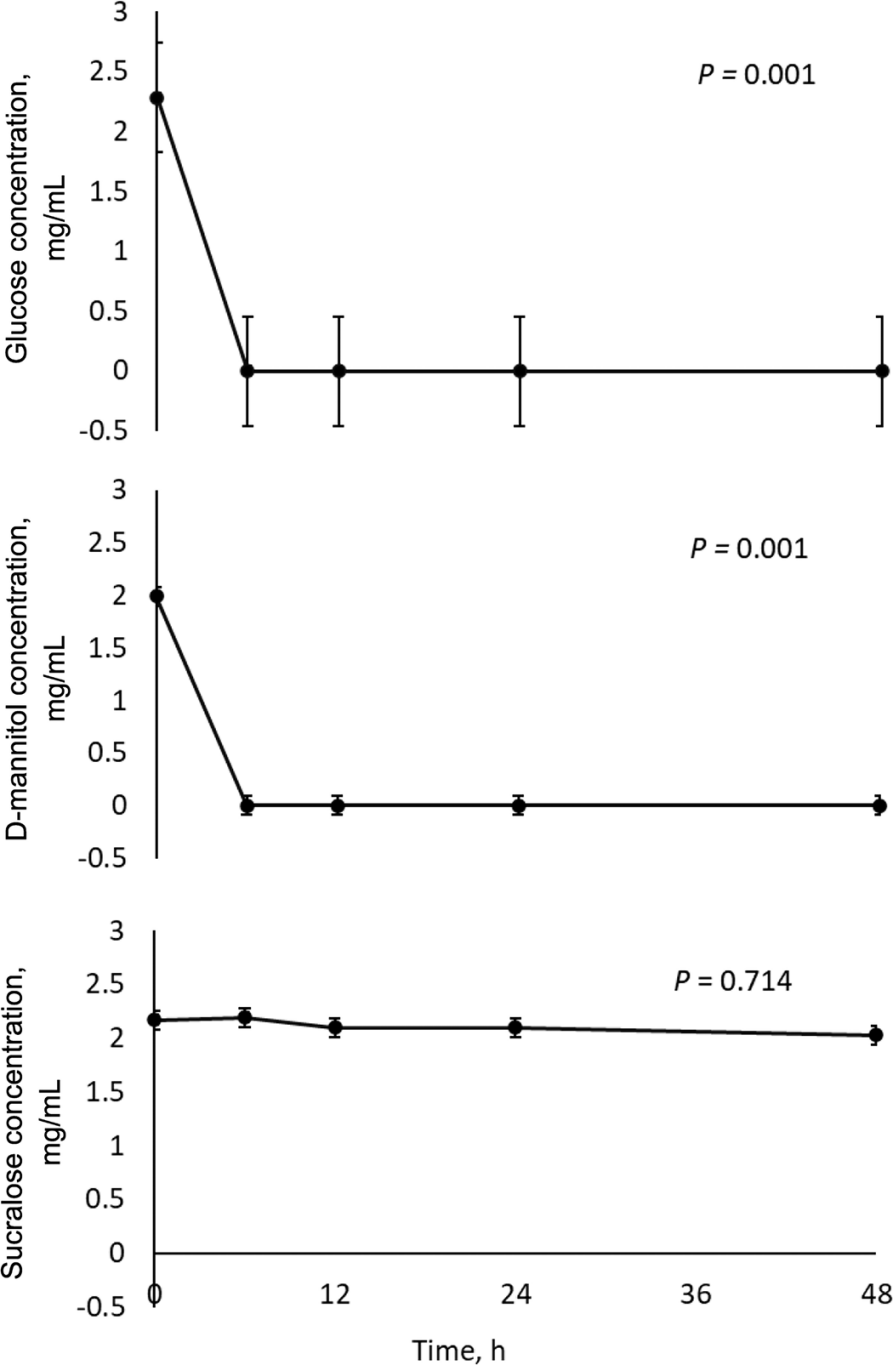
Concentrations of d-glucose, d-mannitol, and sucralose (mg/mL) over time, measured by HPLC-MS, in a closed in vitro rumen culture system. Each point represents the mean of 3 experimental replicates ± SEM.

As expected, our positive methodological control of glucose decreased to negligible concentrations by 6 h of in vitro incubation (*P* = 0.001). d-Mannitol followed the same pattern as glucose (Figure 3; *P* = 0.001), which was different from our hypothesis. Our interpretation is that d-mannitol is degraded in the in vitro rumen culture system and, by extension, is therefore not a viable choice to use in in vivo intestinal permeability tests in adult ruminants when dosed orally. This is a different suggestion than that made by Ahmed et al. (2013), who reported that mannitol seems to largely escape rumen fermentation and therefore might be a viable gut permeability marker in adult ruminants. Notably, Ahmed et al. (2013) did not directly quantify d-mannitol by liquid chromatography as we did; they measured in vitro gas production and VFA concentrations over time after addition of d-mannitol to a closed fermentation system. Our method is arguably more accurate. Our main finding is that we report no change in sucralose concentration over 48 h of incubation in a closed in vitro rumen fermentation system (Figure 3; *P* = 0.714). This is in agreement with similar work reported by Ahmed et al. (2013). Thus, our work along with that of Ahmed et al. (2013), provides sound rationale for further examination of sucralose as an orally dosed rumen-inert sugar in adult ruminants.

## References

[bib1] Ahmed S., Minuti A., Bani P. (2013). *In vitro* rumen fermentation characteristics of some naturally occurring and synthetic sugars. Ital. J. Anim. Sci..

[bib2] Amado L., Berends H., Leal L.N., Wilms J., Van Laar H., Gerrits W.J.J., Martín-Tereso J. (2019). Effect of energy source in calf milk replacer on performance, digestibility, and gut permeability in rearing calves. J. Dairy Sci..

[bib3] Ankom Procedures (2020). *In vitro* True Digestibility Using the DaisyII Incubator. https://www.ankom.com/sites/default/files/document-files/Method_3_Invitro_D200_D200I.pdf.

[bib4] Araujo G., Yunta C., Terré M., Mereu A., Ipharraguerre I., Bach A. (2015). Intestinal permeability and incidence of diarrhea in newborn calves. J. Dairy Sci..

[bib5] Ferreira G., Mertens D.R. (2007). Measuring detergent fibre and insoluble protein in corn silage using crucibles or filter bags. Anim. Feed Sci. Technol..

[bib6] Klein P., Moravcová J., Kleinová T., Volek Z., Skřivanová V. (2007). Assessment of intestinal permeability in preruminant calves by lactulose/mannitol test. J. Anim. Feed Sci..

[bib7] Oba M., Allen M.S. (2000). Effects of brown midrib 3 mutation in corn silage on productivity of dairy cows fed two concentrations of dietary neutral detergent fiber: 2. Chewing activities. J. Dairy Sci..

[bib8] Rao A.S., Camilleri M., Eckert D.J., Busciglio I., Burton D.D., Ryks M., Wong B.S., Lamsam J., Singh R., Zinsmeister A.R. (2011). Urine sugars for in vivo gut permeability: validation and comparisons in irritable bowel syndrome-diarrhea and controls. Am. J. Physiol. Gastrointest. Liver Physiol..

[bib9] Voelker J.A., Allen M.S. (2003). Pelleted beet pulp substituted for high-moisture corn: 1. Effects on feed intake, chewing behavior, and milk production of lactating dairy cows. J. Dairy Sci..

[bib10] Wijtten P.J.A., Verstijnen J.J., van Kempen T.A.T.G., Perdok H.B., Gort G., Verstegen M.W.A. (2011). Lactulose as a marker of intestinal barrier function in pigs after weaning. J. Anim. Sci..

